# Supplementary figures and images for: Diagnostic Accuracy of Procalcitonin upon Emergency Department Admission during SARS-CoV-2 Pandemic
Source: Antibiotics (Basel). 2022 Aug 23;11(9):1141. doi: 10.3390/antibiotics11091141 (PMC9495046; doi:10.3390/antibiotics11091141)

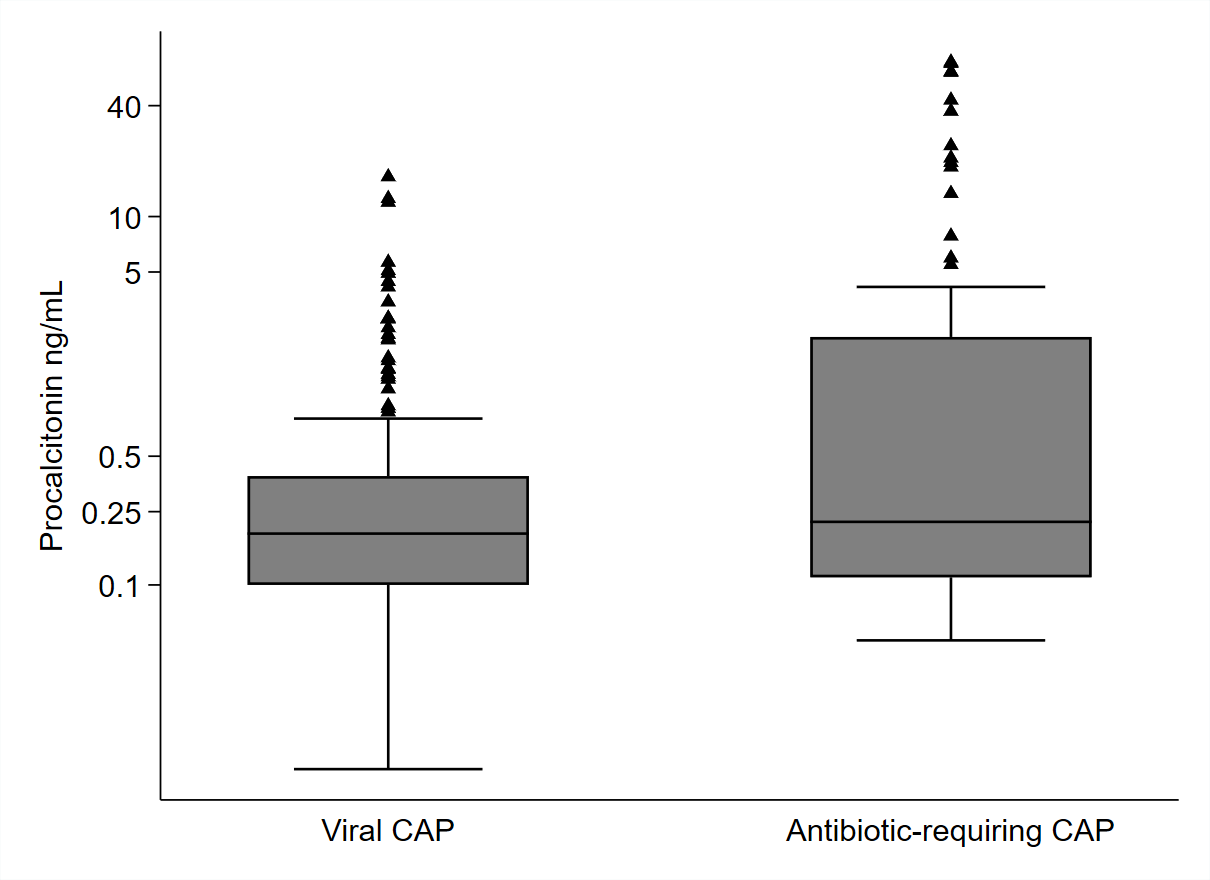

Supplement: Supplementary file 1 [file antibiotics-11-01141-s001.zip › Supplementary Figure S1.tif]
